# *In vitro* assessment for the probiotic potential of *Pichia kudriavzevii*

**DOI:** 10.6026/97320630019441

**Published:** 2023-04-30

**Authors:** Swaruparani Ganapathiwar, Bhima Bhukya

**Affiliations:** 1 Centre for Microbial and Fermentation Technology, Department of Microbiology, University College of Science, Osmania University, Hyderabad 500007, Telangana State, India

**Keywords:** Probiotic yeast, enzyme, phytase, *Pichia Kudriavzevii*

## Abstract

It is of interest to isolate the probiotic yeast *Pichia kudriavzevii* based on its probiotic characteristics and enzyme production. The isolate was able to withstand high acid, bile concentration and showed a high viability. Additionally, it showed auto aggregation ability that increases with time and hydrophobicity with xylene. It was resistant to different antibiotics and showed no hemolytic activity. The isolate was also capable of producing phytase that can break down phytate. Overall, the characteristics of *P. kudriavzevii* suggest that it could potentially have probiotic properties, and its ability to produce phytase could also make it useful in feed and animal industries.

## Background:

In recent times, the poultry industry has emerged to be among the fastest-expanding agro-business sectors in India. Feed costs alone represent approximately 70% of overall poultry production [1]. Because of the world's consistently rising population, the demand for animal products has increased, leading to the use of antibiotic growth promoters [2]. Nevertheless, a growing understanding of the possible risks linked with antibiotic usage has resulted in the development of alternative solutions. To accomplish this, scientists have investigated numerous ways to increase the quantity, quality, and uniformity of farm animals and the products they produce. One such strategy is to incorporate probiotics in the poultry feed, either separately or in combination [3]. Probiotics are beneficial live microorganisms, when supplied in sufficient amounts promote the host's health. [4, 5]. Poultry feed has a high concentration of protein and carbohydrates, along with anti-nutrients such as phytic acid and polyphenol. Soybean and corn are two important feed ingredients in poultry diets across the world, and they contain anti-nutritive components (such as protease and NSP inhibitors) in varying levels that can restrict proper digestion and nutrient absorption in the gastrointestinal system [6]. To counteract the detrimental impacts of anti-nutritional components, exogenous feed enzymes must be added to an animal feed that increases the nutrient quality of the feed components [7].

Enzymes are currently used in animal feed to boost their nutritional value. Feed along with probiotic organisms not only improves the nutrient profile but also exerts beneficial health effects. Phytases are enzymes that break down phytic acid into a series of phosphate esters of Myo-inositol and phosphate [8]. These enzymes are found naturally in the digestive tracts of some animals, microorganisms and some plants. Phytases are commonly used as feed additives in livestock to improve nutrient utilization and reduce environmental pollution. The addition of phytase into animal diet reduces the need for extra phosphorus supplements [9]. Therefore, it is of interest to assess the probiotic properties and enzyme profiling of *P. kudriavzevii* which was isolated from a chicken intestine.

## Methodology:

## Sample collection and Isolation:

A total 25 samples of toddy, soil from poultry form, and chicken intestine were collected from different regions of Hyderabad, Telangana, India. Samples were collected in sterile zipper-lock bags followed by serial dilution using distilled (or saline) water.100 µl was spread onto YPD (Hi media) agar plates and incubated at 30°C for 48 hrs [10]. Yeast isolates were selected based on their cultural, morphological features, growth patterns, and microscopic examination. The selected isolates were sub cultured on YPD agar slants and stored at 4°C in the refrigerator for further analysis.

## Enzyme production:

Selected probiotic yeast isolates were evaluated for their enzyme production (phytase). Phytase screening medium (PSM- Sodium phytate-5g, glucose-15g, ammonium nitrate-5g, magnesium sulphate-0.5g, potassium chloride- 0.5g, ferrous sulphate-0.01g, manganese sulphate-0.01g, pH-6.0) was used for qualitative analysis. Selected isolates were spot inoculated on PSM agar plates and incubated for 48 hrs at 30°C [11]. The diameter of the zone around the colony was measured and correlated with enzyme activity.

## Acid and Bile Tolerance:

From above, selected yeast isolates were inoculated in YPD (Hi-media) broth and incubated for 24 hrs. After incubation, culture was centrifuged, washed with PBS and then the pellet was dissolved in 3ml PBS buffer with pH 2 and 3. The suspensions were incubated for about 4 hrs and then inoculated on YPD agar plates. Viable cells were calculated by the standard plate count method and expressed as survival rate. Bile salt tolerance was performed with activated overnight cultures, centrifuged and added to the buffer with 0.1, 0.5, and 1% ox bile. Bile tolerance was tested after 4 hrs of incubation by counting viable cells using the standard plate count method [12]. The data on each parameter was statistically subjected to analysis of variance (ANOVA) using Tukey's post-hoc one way analysis and significance was considered at *p* < 0.01.

## Auto-aggregation property:

The auto-aggregation ability of probiotics is associated with the adhesion ability of microorganisms. Selected yeast isolate was activated at 30°C and the cells were harvested by centrifugation at 7000 rpm for 5 mins. The pellet was washed twice and re-suspended in PBS. The cell suspensions have mixed by vortex for 10 mins, determined the autoaggregation at 3 hrs and 20 hrs by collecting the upper suspension, and absorbance had measured at 600nm [13].

The auto aggregation percentage was expressed as 1-(At/A0) X100.

Where At represents the absorbance at 3hrs and 20 hrs, A0 denotes the absorbance at time 0

## Cell surface Hydrophobicity:

Overnight cultures were harvested by centrifugation at 7000rpm for 5 mins, and the pellet was washed twice and re-suspended in 5 ml of PBS. The cell suspension was allowed in contact with xylene. The cells were vortexed for 3 mins. The suspension was undisturbed for 10 mins, the aqueous phase was removed and the absorbance was measured at 600nm [13].

The cell surface hydrophobicity was calculated = (OD initial-OD final)/OD initial X 100

## Safety assessment:

The hemolytic activity was determined by inoculating the yeast isolate on blood agar plates containing sheep blood and incubated for 48 h at 30°C. The development of a clear zone around the colony indicates a positive result [14]. Antibiotic resistance of the yeast isolate was performed by the disc diffusion method using different antibiotic discs such as Nystatin (50µg), Miconazole (10 µg), Clotrimazole (10 µg), Fluconazole (10µg), Ketoconazole (50 µg), Itraconazole (10 µg), Cefmetazole (10 µg) (Sigma Aldrich).The antibiotic discs were placed on yeast-inoculated YPD agar plates and incubated for 48 h at 30°C, and a zone of inhibition was observed [15].

## Phytase assay:

10 µl extracted enzyme sample was mixed with 40 µl 0.1M sodium acetate buffer (0.2M acetic acid and 0.2M sodium acetate, pH 5.0), containing phytic acid dipotassium salt (final phytic acid concentration 2 g/l) and inncubated the samples for 30 mins at 30°C. After incubation, 50µL of 10% trichloracetic acid had added to stop the reaction. Determination of liberated phosphate was estimated by adding 800 µL acid molybdate reagent (1 volume of 10mM ammonium heptamolybdat-tetrahydrate, 1 volume of 2.5M sulphuric acid, and 2 volumes of acetone). After that, OD355nm was measured using sodium acetate buffer and trichloracetic acid as blank. Phytase activity is defined as the amount of enzyme releasing 1µM inorganic phosphate/ml under standard assay conditions [11].

## Molecular Identification:

Based on probiotic characteristics and enzyme production, the yeast isolate TS2 was characterized by 5.8S rRNA sequencing by submitting to Macrogen Inc. South Korea. The obtained sequences were blast searched and submitted to NCBI for GenBank accession number. A phylogenetic tree has constructed with MEGA X software. The evolutionary distances were inferred using the Maximum Likelihood method and the Tamura-Nei model [16].

## Results and Discussion:

Probiotics produce a variety of valuable enzymes that improve animal health by assisting in food digestion. In addition, several studies have shown that adding amylases, xylanase, and phytase can reduce the adverse effects of anti-nutritional factors in monogastric animals fed with different raw materials [17]. A total of 25 yeast strains were isolated from various samples collected from different places. Ten yeast isolates were selected based on differential growth patterns on agar medium. The microscopic examination further confirmed them and from these, only two yeast isolates were able to produce phytase on PSM agar.

## Probiotic Features:

For probiotic strains to be viable and feasible in the intestine, acid tolerance is a fundamental characteristic that has to be considered [18]. Acid tolerance in probiotics is essential to withstand gastric stress to use them as dietary supplements. Table 1 shows the survival rate of yeast isolates in different acid and bile conditions. When these isolates were exposed, TS2 had able to tolerate these conditions with more than 50%, whereas SB2 could not withstand the above conditions. When exposed to bile salt, they showed a tolerance of up to 1% and TS2 showed the highest tolerance compared to the other isolate. The two isolates tolerate high pH and bile, following Rajkowska *et al*. and Srinivas *et al*. [12, 15]. The selected isolates showed tolerance to bile salt at 1%. Helmy *et al*. [19] observed probiotic yeast *P. kudriavzeviil* QLB isolated from Karish cheese showed tolerance to the bile concentration of 2%. Probiotic microbe colonization also enhances host defense mechanism and hinders the entry of pathogens into the digestive tract. After 3 hrs of incubation, isolate TS2 exhibited above 50% auto aggregation, which increased to 85% after 20 hrs. A similar result was observed by Lata *et al*. [14] where P kudrivenzii isolated from mango pickle showed 87% auto aggregation after 24 hrs. High hydrophobicity-exhibiting strains have good adhesion properties to intestinal cells. Yeast isolate showed 82% hydrophobicity with xylene after 20 hrs of incubation and similar result was reported by Hossain *et al*. [20].

## Enzyme production:

When feed is supplemented with phytase, it degrades the phytate, and the bound minerals are released, thereby increasing the availability of the minerals for absorption. Thus the nutrient value of the food or feed is improved along with promoting the essential dietary minerals bioavailability [21]. Yeast isolates TS2 showed the highest phytase production of 34 U/ml. Ogunremi, [22] and Syal *et al*. [13] also observed phytase production from *p. kudrivenzii*.

## Safety Assessmentcri:

In the present study, there was no evidence of hemolysis identified. Isolate TS2 was identified as non-pathogenic, indicating that it is safe to use as a probiotic. The antibiotic sensitivity was assessed by the disc diffusion method with various commercial antifungals. TS2 was resistant to Nystatin, Miconazole, Fluconazole, and sensitive Clotrimazole (10 µg), Itraconazole (10 µg), Ketoconazole (50 µg), and Cefmetazole (10 µg).

##  Identification:

**Figure 1:**
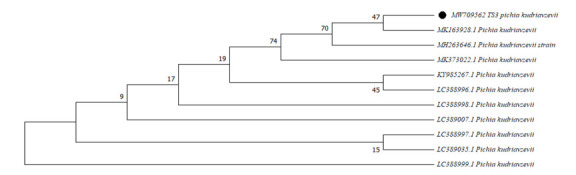
Phylogenic dendrogramof *Pichia Kudrivzevii* showing relationship between isolates. Evolutionary analysis was performed in MEGA X.

Yeast isolate TS2 was selected based on probiotic characterization. The isolate showed 94% similarity with *Pichia kudivenzii* when matched with NCBI database sequences. The sequence was deposited in the NCBI GenBank data library and obtained MW709562 accession number. The phylogenetic tree was constructed with Neighbor-Joining method and evolutionary distances were calculated using the Maximum Composite Likelihood method (Figure 1).

## Conclusion:

Data shows that *P. kudriavzeviil*, isolated from the chicken intestine, exhibits promising characteristics that make it an excellent probiotic candidate. Furthermore, *P. kudriavzeviil* TS2 has the potential to degrade phytic acid, which could also promote dietary nutrition. This property suggests it is a suitable probiotic candidate to employ as a food and feed supplement. Altogether, the findings show that *P. kudriavzeviil* possesses beneficial properties that make it a promising probiotic candidate.

## Figures and Tables

**Table 1:** pH and bile tolerance of yeast isolates (Survival rate)

**S.No**	**Isolates**	**pH tolerance **		** Bile tolerance**		
		pH 2	pH 3	0.10%	0.50%	1%
1	TS2	74.45±1.90	78.85±1.66	90.56±1.53	74.33±0.79	61.27±1.06
2	Sc2	61.31±2.61	63.23±1.00	73.78±1.79	61.29±1.10	43.01±2.56
3	Td4	43.15±3.47	46.04±1.54	59.56±1.53	55.70±2.74	36.38±1.00
4	Sb	23.71±2.71	22.30±1.06	44.96±1.58	41.56±1.53	30.89±1.68
